# 

**Published:** 1877-12-20

**Authors:** 


☞All communications relating to the business of The Record, for the year 1877, must be addressed to

DR. R. C. WORD,
Business Manager Southern Med. Rec.,
Atlanta, Ga.

☞Brief and practical communications are solicited on all subjects pertaining to medicine, also reports of cases in practice.

☞ Send money by check, postal order or registered letter.

☞Write your name, post-office, county and State plainly.

☞ In the notice in November number of the drug firm of Pemberton, Samuels & Reynolds, the name of the first party, instead of J. B., should have been put J. S. Pemberton.

Receipted for 1877.—Drs. John Coston, A. H. Peek, R. L. Key, J. P. Rice, T. J. Fout, T. L. Darby, J. W. Huff, T. L. Quillan, '77 and '78.

### INDEX.

A

Abortion, black haw in.................... 181
Absorption of iodine by the skin........... 22
Accacia, mucilage of....................... 22
Acid deposit in urine...................... 161
Acne, treatment of ...................223 43
Aconite ................................... 144
Aconite in headache........................ 105
Acute amoniacle collapse................... 249
After pains............................50, 191
Ague pill.................................. 190
Air, quantity inhaled...................... 134
Air in the ground.......................... 193
Alabama, Medical Association of............ 137
Albumen, in consumption.................... 185
Albumen, nitric acid test for.............. 223
Albuminuria, treatment of.................. 9
Albuminuria scarlatinal.................... 14
Albuminuria, arsenic in...............273, 330
Albuminate of iron in anæmia............... 245
Alcohol, to relieve thirst for............. 242
Alcohol, as an abortive in diptheria....... 304
Alcoholic headache......................... 35
Alitura wine............................... 193
Alpha fibre................................ 24
Alterative for pharyngitis................. 79
Amenorrhea in anæmic subjects.............. 105
American Medical Association......165, 109, 195
American Pharmaceutical Association...282, 304
Ammonia in bronchial and lung troubles..... 59
Ammonia treatment of rheumatism............ 258
Amputation, hip joint,..................... 13
Amputation by elastic ligature,............ 269
Amputation treated antiseptically.......... 10
Anæmic headache ........................... 36
Anaemia treated by transfusion............. 154
Anaesthesia, difference of ether and chloroform........ 39
Anaesthesia................................ 102
Anaesthesia, who discovered by............. 184
Anaesthesia, from bromohydrate of quinia... 183
Anaesthetic, bromide of ethyl as........... 214
Anasarca, chinopodium in................... 329
Anaesthetic agent new...................... 75
Anaesthetic, eau de cologne as............. 163
Anaesthetic mixture........................ 163
Anderson, Dr. L. B, on constipation........ 112
Antibilous laxative pill................... 77
Antibilous purgative powder................ 80
Angina pectoris............................ 162
Antihemorrhagic............................ 280
Anthrax.................................... 290
Antihydropin..........................75, 193
Antiemetic................................. 134
Antiseptic treatment of thigh.............. 10
Antiseptic method, in ovariotomy........... 16
Antiseptic sedative........................ 222
Antiseptic surgery......................... 294
Antiseptic vinegar......................... 275
Antispasmodic and nervine.................. 23
Apocynum canabinum in dropsy............... 151
Apthous ulcerations........................ 160
Arnica poisonous........................... 251
Arkansas Medical Association............... 109
Aromatic syrup of galls.................... 190
Arsenic, antidotes for.................329, 78
Arsenic in chronic chills.................. 250
Arsenic in Albuminuria................330, 273
Arsenic in the Stomach..................... 82
Arrowroot jelly............................ 187
Arthritis rheumatic........................ 124
Arundell eye glasses....................... 26
Ascarides vermicularis..................... 159
Ascites.................................... 76
Ascites, copaiba in........................ 334
Asthma spasmodic........................... 129
Asthma, spasmodic, syrup for............... 4
Asthma, secret remedy for.................. 126
Asthma, smoking belladonna for............. 48
Asthmatic paroxysm......................... 79
Asthmatic paroxysms, in intervals.......... 79
Asthma jaborandi in........................ 241
Asthma and bronchitis...................... 250
Assafoetida, syrupus....................... 4
Atropia, antidote to hydrocyanic acid...... 13
Atropia poisoning.......................... 48
Atropia with morphia subcutaneously........ 242

B

Bacteria................................... 132
Baking powder.............................. 100
Baldness................................... 191
Balsamic mixture, LaFayette's.............. 79
Balsam of tolu, for wounds................. 182
Bandages for ulcers of leg................. 150
Bathing, cool, for summer complaints....... 41
Baths, stringent and tonic................. 273
Barry, Dr. W. D., address.................. 137
Bed Sores.................................. 335
Beer, ginger............................... 106
Beef-tea................................... 187
Beef-tea and cream enema................... 251
Belladonna................................. 147
Belladonna in constipation................. 275
Belladonna in asthma....................... 48
Belladonna in dysentery.................... 234
Benzoate of lithium in gout................ 241
Benzoic acid, in cystitis.............193, 194
Bicarbonate of Soda........................ 278
Bile, agents affecting secretion of........ 103
Billious attacks........................... 183
Bismuth, subnitrate of in diarrhea......... 190
Births, plural............................. 107
Bisulphide of carbon....................... 52
Blacking, for stove........................ 106
Blacking, for harness...................... 107
Bladder, catarrh of........................ 106
Bladder affections, cocklebur in........... 124
Black haw in abortion, &c.................. 181
Blenorrhœa, corrosive sublimate............ 81

Blenorrhagic epidydimus, iodoform in...... 277
Bleeding at the cord.... 31
Blister, to lessen pain of.... 128
Bloodless operation, new. .... 99
Blue glass cure... .... 137
Board of health......... ..............26, 55
Boils and carbuncles.... 335
Boldo.... 22
Book Notices....
27, 56, 84, 110, 138, 226, 196, 254, 282, 310, 339
Bogies, medicated.... 22
Brain, softening of.... 228
Brain, circulation of, experiments on........ 336
Breast bandaging of.... 21
Brights disease, dignostic symtoms of...... 126
Bristow, Dr. J. R., scraps of practice....... 307
Bromohydrate of cicuta.... 126
Bromohydrate of quinia.... 13
Bromohydrate of conia.... 132
Bromohydrate of quinia, hypodermically ... 183
Bromide of ammonium.... 147
Bromide of iron .... 223
Bromide of arsenic, in epilepsy... .... 42
Bromide of ethyl, as an anæsthetic.... 214
Bromide of potassium, rash from.... 243
Bromide of potassium, in hemmorrhage... . 82
Bromide of potassium, as a caustic.... 20
Bruises, application for.... 106
Bronchitis.... ....307 68
Bronchial and lung troubles, ammonia in ... 59
Bronchial and lung troubles, potion for..... 77
Bronchihl catarrh, carbolic spray in........ 76
Bronchitis and asthma .. .... 250
Bronchitis of children..... .... 270
Burns, application for.....333 106
Buboes, cold douche for.... 77
Button, vomited after two years.... 51
Butty-chloral, action of.... 218

C

Cactus...... .... 148
Calcium, a compound.... 252
Calomel, saccharated.... 80
Camphor, carbolized .... 100
Cancer.... 151
Cancer, epithelial.... 233
Canada balsam.... 334
Carbuncles and boils.... 330
Carbolic acid spray... .... 335
Carbolic acid, antidote for.... 102
Carbolic camphor, observations on......... 100
Carbolic collodion .... 221
Carbolic spray, in bronchial catarrh........ 76
Carbonate of Ammonia, in snake bite....... 223
Carbonate of iodine.... 23
Carbuncle.... 255
Carbuncle, treatment of... .....120 205
Castor oil .... 50
Castor oil, to take without tasting.... 333
Cascar bark.... 163
Cataract, modified operation for.... 122
Catarrh, intestinal, of infants.... 131
Catarrh, nasal.... 17
Catarrh, nasal, new method of treating.... 217
Catarrh, naso pharyngial.... 128
Catarrh of the bladder.... 106
Catarrh Remedy, Sage's .... 126
Catarrh snuff.... .... 221
Catheterism, morphine in.... 330
Cathartic pills, improved .... 79
Celluloid.... .... 184
Cerebral hyperæmia, hydrobromic acid in... 306
Cerebral neuralgia.... .. 51
Cerebro spinal meningitis............29 111 191
Chamberlain's relief, formula of .... 12
Chicken broth .... 187
Chilblains... .... 107
Chills, chronic .... ............152 218 274 250
Chills, remedy for.... .... 249
Chill, to abort.... ... 249
Chill tonic.... .... 105
Chills, salacine for... .... 189
Chinipodium anthelminticum, in anasarca... 329
Chloral.... 277
Chloral cream.... 191
Chloral and alcohol... .... ... 333
Chloral in tetanus.... 105
Chloral, hydrate, uses of.... .... 273
Chloral in convulsions.... .... 216
Chloral, in eclampsia.... 220
Chloral, hydrate, in diabetes.... 67
Chloral and opium, distinction between...... 99
Chloral plaster.... .... 78
Chloral in ulcers.... .... 50
Chloral, small doses in delirium tremens ... 81
Chloral drinker.... 392
Chloral, locally, in neuralgia.... 159
Chloral for false membrane. .... 160
Chloroform, spirits of.... .... 335
Chloroform, Hartshorn's elixir of.... 334
Chloroform, internal administration of...... 19
Chloroform, Venus pulse from.... .... 52
Chloroform, death from.... 93
Chloroform, experiments on .... 45
Chloroform and ether, difference of.... .... 39
Chloroform, deep injection of in sciatica. .. 127
Chloroform, use of, in labor.... .........206 129
Chloroform, emulsion.... .... 191
Chloroform as a linament.... 162
Cholera morbus.........163, 97
Cholera, infantum, vomiting in.... 180
Cholera, infantum.... 157
Cholera, infantum, monobromide of camphor in .... 226
Chorea.... .........247 4
Chorea, sulphate of zinc in.... .... 160
Chronic malarial toxemia.... 274
Cicutine, bromhydrate of.... 126
Cider, to keep sweet.... .... 107
Cinchonidia.... .........257 110
Cinchonidia and cincho quinine.... 282
Cincho-quinine.........167 249
Citrate of caffiene .. .... 191
Climate of Upper Georgia.... 55
Coca.........217 152
Coclebur.... .... 124
Cod liver oil and bark enema.... 251
Coffee leaf.. .... 22
Cold douche, etc., in consumption, for babies.........77 41
Cold water subcutaneously in rheumatism.. 76
Cold baths, in infantile diarrhea.... 127
Cold in the head.... .... 250
Cold feet.... .... 302
Colic.... 192
Colic, hepatic, podophyllin for.... 276
Colic infantile.... .... 221
Coliquitive sweating.... 74
Collodion flexile, in eczema.... .... 197
Collinsonia.... 147

Colored spectacles........................ 77
Cologne water.............................. 107
Colocynth, for abdominal pains............... 300
Columbæ, pulvis compositis................ 4
Compressed air, new instrument for using... 298
Congestive chill.............................. 189
Conium maculatum......................50 251
Conia, bromohydrate of.................... 132
Constipation............................180 187 112
Constipation, belladonna in................ 275
Consultations...........................2[illegible]5 166
Consumption, cold douche in.............. 44
Consumption, albumen in.................. 185
Convulsions of children.................... 228
Convulsions, chloral in....................216 235
Copaiba in ascites.......................... 334
Corneal ulcer................................ 159
Cosmoline.................................... 276
Cough, remedies for.....................106 279 146
Cough, Ricord's pills for.................. 334
Country physicians......................... 109
Cows' milk for babies...................... 130
Coxalgia...................................... 101
Cracked skin................................. 10
Cramp, nocturnal........................... 238
Craniotomy................................... 209
Creosote, by spray.......................... 50
Chromic acid, in ulceration of os........... 131
Chromic acid, for warts.................... 77
Croup, kerosene oil in..................... 160
Crysarobin, what is it?..................... 248
Croup, eucalyptus in....................... 215
Croup, membraneous, new treatment........ 255
Crysophanic acid........................... 334
Current neurological matters.............[illegible] 201
Cutaneous affections, petroleum in........ 14
Cutaneous secretions, effect of suppressing....................................275 218
Cyanide of zinc in rheumatism............. 305
Cestytes, benzoic acid in.................. 183 19[illegible]

D

Davyum...................................... 224
Deaf and dumb.............................. 82
Death from want of salt.................... 279
Definitions.................................. 56
Delirium tremens........................... 250
Delirium tremens, chloral in.............. 81
Dentifrice................................... 106
Dentition, first.............................. 203
Dental irritation............................ 237
Diabetes melitus, chloral hydrate in........ 67
Diabetes melitus, chloral hydrate in........ 57
Diabetes.....................................134 276
Dialyzed iron............................... 191
Diarrhea, autumnal......................... 272
Diarrhea, carbolic acid in.................. 333
Diarrhea chronic, treatment of............ 71
Diarrhea Infantile, cold baths in..........180 127
Diarrhea, mixture Velpean's............... 23
Diarrhea, obstinate......................... 163
Diarrhea, chronic R for.................... 224
Diarrhea, simple............................ 190
Diascoria villosa, or wild yam............. 152
Diet for infants............................. 222
Diatetic preparations....................... 187
Dilatation of the cervix, gelseminum for..... 5
Dilatation of the uterus.................... 70
Diphtheria..................................223 153
Diphtheria, treatment of.................. 39
Diphtheria, select formula for.............. 30
Diphtheria, gargle for...................... 280
Diphtheria, formula for..................... 77
Diphtheria, vesical, disturbance from...... 14
Diseases of the skin, Prof. During on....... 84
Disinfectant for privies.................... 223
Doses for children.......................... 279
Donald, Dr. A. on materialistic psychology. 203
Dropsical affections, apocynum c, in........ 151
Dropsical and albuminous conditions...... 226
Drosera...................................... 147
Dropsical effusions......................... 17
Dugas' method of taxis..................... 123
Dyer, Dr. Geo. A., on maternal impressions.. 228
Dyer, Dr. Geo. A. on cerebro-spinal meningitis....................................... 29
Dyer, Dr. Geo. A. on inguinal hernia........ 30
Dynamite.................................... 82
Dying, duty of the Doctor to the.......... 316
Dsymenorrhea, black haw in............... 181
Dysmenorrhea, hysterical.................. 191
Dysmenorrhea, remedy for................23 95
Dysmenorrhea, hydrastis in................ 232
Dysmenorrhea, prescriptions for.......... 249
Dysmenorrhea, wet blanket pack for....... 174
Dysentery...................................158 191
Dysentery, chronic, topical treatment of...15 65
Dysentery, belladonna in.................. 234
Dysentery, warm injections in............. 20
Dysentery ipecac in........................ 159
Dyspesia, nervous, hydrobromic acid in..... 307
Dyspepsia, formula for..................... 94
Dyspepsia and cardialgia.................. 105
Dyspepsia, some forms of.................. 6
Dyspepsia, pyrosis.......................... 122
Dyspeptic headache........................ 35

E

Ear diseases, iodoform in.................. 276
Ear, foreign bodies in...................... 260
Eau de Cologne as an anaesthetic.......... 104
Ecbolic action of quinine.................. 217
Eczema......................................54 188
Eczema, chronic............................ 72
Eczena, collodion flexile in................ 297
Eclampria, peurperal....................... 121
Eclampsia, chloral in...................... 220
Egg brandy.................................. 280
Elastic ligature, amputation by............ 269
Electricity in herpes zoster................ 132
Electric plant............................... 336
Electrolysis................................. 195
Elixir of glycerine.......................... 150
Emenagogue pill............................ 76
Emulsion of phosphorated oil.............. 48
Emulsion of phosphorus................... 79
Enema nutritive............................ 250
Enteric fever, is it contagious?............ 212
Epilepsy....................................32 12
Epilepsy, bromide of arsenic for........... 42
Epilepsy, Hydrobromic acid in............. 306
Epilepsy, santonine in..................... 224
Epileptiform neuralgia..................... 222
Epileptic seizures at the menstrual period.. 51
Epileptics, vegetable diet for.............. 50
Epistaxis, simple mode of checking........ 242
Epithelial cancer of lips and face.......... 233
Epizoot...................................... 110
Ergot in typhoid fever.....................44 76
Eruptive diseases of the scalp............. 80

Erysipelas and vaccination.............. 332
Esophagus, foreign bodies in.............. 276
Ether hydrobromic.... .................... 107
Encalyptus as a disinfectant, &c........... 7
Encalyptus in neuralgia.................. 7
Encalyptus in croup ...................... 215
Eve, Dr. Paul F, death of.................. 309
Evolution.................................. 134
Exopthmalmic goiter...................... 18
Extract of logwood as a disinfectant ....... 126
Eye-glasses, Arundell...................... 26
Eyes, sore, remedy for... ................. 279
Eyes, hygiene of......................... 291
Eye-water, Thompson's. ................... 221

F

False membrane, chloral for.............. 180
Fauces, remedies for...................... 146
Fetid feet................................. 77
Feet, cold ................................ 302
Fetus in utero, effects of medicine upon..... 153
Feeling the pulse by telegraph............ 252
Femur, dislocation of, to reduce........... 292
Femur, fracture of......................... 318
Female chemist.......................331 327
Fever and ague........................... 277
Finger, value of.......................... 52
First intention, union by................. 148
First dentition............................ 203
Fire ...................................... 308
Flags, relief, formula of.................. 12
Flaxseed lemonade........................ 188
Flaxseed tea.............................. 222
Flatulent or rum stomach... .............. 334
Flies, protection against.................. 242
Fluid extract of kidney leaf com.........226 199
Folicular pharingitis..................... 134
Forceps, obstetrical use of................ 183
Foreign bodies in esophagus.............. 276
Fracture, when to dress.................. 80
French, Dr. Geo. F., respiratory brace...... 266
Freckles .................................. 333
Fucus vesiculosus......................... 154
Fumigation in syphilis.................... 314

G

Gall stones, inspection of................ 186
Galls, aromatic syrup of.................. 190
Gallic acid in phthises.................... 162
Gallactagogue, jaborandi, as.............21, 16
Gargle, tannin............................ 336
Gargle, for foul ulcers................... 336
Gargle for diptheria...................... 280
Gasoline.................................. 107
Gastric juices, properties of.............. 216
Gastro elytrotomy........................ 241
Gastric ulcer, milk treatment of.......... 47
Gelseminum.................113, 114, 62, 207 286
Gelseminnm in neuralgia................94 228
Gelseminum and quinia.................... 328
Gelseminum for dilatation of cervix........ 4
[illegible] mi num as antidate for veratrum....... 40
[illegible] a na quinque flora...................... 182
Georgia Medical Association.............. 135
Giant powder............................. 82
Ginger beer............................... 106
Glottis, spasm of......................... 183
Glycente of kephaline..................... 142
Glycyrrhizæ, elixir of.................... 150
Glycerine a poison........................ 154
Glycerine, solvent for phosphorus......... .. 22
Goelet, Dr. A. H., of N. Y., on management of labor.............................. 288
Goelet, Dr. A. H., of N. Y., on retroflection of uterus............................ 57
Goiter exopthalmic........................ 18
Gold, maleability of....................... 134
Gonalgia, or hysterical arthralgia.......... 247
Gonorrhea ..............................250 191
Gonnorrhea and gleet.. ................... 81
Gordon, W. A., M. D., on hydrastis........ 232
Goss, Dr. I. J. M., on gelseminum.......113 286
Goss, Dr. I. J. M., on aconite.............. 144
Gout, benzoate of lithium in............... 241
Gout, headache from...................... 34
Granulated lids........................... 190
Green, Dr. W. A., of Georgia, on pharmaceutical science.......................... 170
Same on dyspepsia apyrosis................ 172
Same on sugar-coated pills................ 198
Same on fluid extract kidney leaf com....... 199
Gregory, Dr. C. L., notes in practice... .... 94
Grindelia................................. 147
Guild, Dr. L. A., hemorrhagic malarial fever 172
Guaranin in migraine.................. .. 239

H.

Hæmaturia................................ 161
Hair, falling, recipe for.............. .... 106
Hair, wash................................ 334
Hamilton, Dr. J. L., dropsical conditions... 226
Harlem oil................................ 23
Harness blacking.......................... 107
Hay fever...............................75 280
Headache, therapeutics of ................. 32
Headache, sick............................ 33
Headache, malarial ....................... 34
Headache, from gout...................... 34
Headache, syphalitic...................... 34
Headache, rheumatic...................... 34
Headache, uræmic......................... 34
Headache, alcoholic...................... 35
Headache, dyspeptic....................... 35
Headache, congestive...................... 36
Headache, anæmic......................... 36
Heart, palpitation, etc. .................. 94
Heat, prickly, to relieve.................. 74
Hemoptysis............................... 251
Hemorrhage, post partem.......275 268 156 13
Hemorrhage, tortion in.................... 15
Hemorrhage, from the cord................ 31
Hemorrhage, visical...................... 131
Hemrrahgic malarial fever.............135 172
Hemorrhoids, dilatation of spincter for..... 47
Hemorrhoids, radical cure of.............. 49
Hen's egg, wonders of..................... 82
Hepatitis, chronic...................161 335
Hernia umbilical, in infants............... 49
Hernia inguinal, case of ................. 30
Hernia, sneezing to reduce................ 95
Herendon, Dr. Z. B., how to take castor oil....................................... 333
Herpes................................... 116
Herpes, phlyctinodes, etc................. 117
Hip joint, amputation..................... 13
Hip joint, reduction of, etc............292 208
Hip joint, diseases of, in children.......... 302
Hoarseness, nitric acid in...............78 224
Hobbs, Dr. A. G., cinchonidia............ 339

Hodge, Dr. G. D., on diptheria.... 30
Horse-shoeing.... 254
Hooping cough....245 105
Hot packing, in rheumatism.... 78
Hunt, Dr. W. G., malformation.... 2
Hydrobromic ether.... 107
Hydrocyanic acid, atropia for.... 13
Hydrobromic acid in prescriptions.... 306
Hydrobromic acid in tinnitus aurium.... 249
Hydrophobia during pregnancy.... 330
Hydrophobia cured by snake bite.... 244
Hydrastis canadensis in uterine hemorrhage.... 232
Hydrate of chloral in convulsions.... 235
Hydrocele.... 156
Hydrogongue cathartic.... 251
Hypophosphite of sodium....335 22 14
Hypophosphites in phthisis....246 162
Hypodermic doses.... 189
Hysteria.... 192
Hysterical dysmenorrhœa.... 192
Hysterica epileptica.... 336

I.

Impotence....72 309
Incontinence of urine....251 223
India rubber solution, in psoriasis.... 17
Indigestion.... 180
Indian mea gruel.... 187
Indian cholagogue, formula for.... 279
Inebriety.... 219
Infants, alimentation of....185 222 271
Inge, Dr. R., tracheotomy for foreign body.. 58
Insanity.... 307
Inhalation, remedies by.... 148
Ingrowing of nails.... 257
Insect destroyer.... 17
Insects, tomato leaves for.... 185
Insomnia.... 153 275
Intermittent fever....23 22 218 190
International regulations.... 55
Intestinal catarrh, of infants, etc....131 271
Iodine inhalations....46 336
Iodine, carbonate of.... 23
Iodine, absorption of by the skin.... 22
Iodine, administered through a nurse,.... 186
Iodine, preparations of....98 242
Iodide of lead poisoning.... 71
Iodoform pencils.... 189
Iodoform, in ear diseases.... 276
Iodoform, in blenorrhagic epididimus.... 277
Iodoform, in typhoid fever.... 332
Ipecacuanha.... 146
Ipecac treatment of dysentery.... 159
Irritable bladder from acid.... 250
Irritable bladder and painful micturition.... 24
Iron, effects of on the blood.... 329
Iron, when not to give.... 304
Iron and alum mass.... 170

J.

Jaborandi.... 212
Jaborandi, as a galactagogue....76 21
Jaborandi in asthma.... 241
Japanese therapeutics.... 329
Jaundice.... 160
Jetties.... 252
Journals, medical.... 1
Junctus acutus, in asthma.... 76
Jupiter, the planet.... 134

K.

Kellog's red drops, formula of.... 12
Kerosene oil in croup.... 160
Kerosene liniment.... 332
King, Dr. F., on cincho quinine.... 167
Kidney affection, cocklebur in.... 124
Knott, Dr. J. J., on yellow fever.... 278
Knott, Dr. J. J., sulphate cinchonidia.... 338

L.

Labor, hints on management of....86 288
Labor delayed, death from.... 64
Lactate of soda, in insomnia, etc.... 224
Lactophosphate of lime to fill teeth.... 188
La Fayette mixture, balsamic.... 79
Lane, Dr. E. W., cerebro spinal meningitis.. 111
Laparatomy.... 155
Lavœsium.... 308
Laxative pill.... 24
Laxative anti-billious pill.... 77
Laxative for piles.... 191
Lead, iodide of, poisoning by.... 71
Leanness, remedy for.... 106
Leather, to glue.... 107
Leeches, to keep.... 336
Leeches, to make bite.... 103
Lemon Juice in rheumatism.... 327
Lemons, to keep.... 106
Lewis, Dr. J. Merriwether, on malarial fevers.... 284
Lewis, Dr. J. Merriwether, ammonia treatment of rheumatism.... 258
Ligamentum patellæ, rupture of.... 266
Lime in the eye.... 333
Lime water.... 107
Liniment, a good one.... 280
Lister's antiseptic method, in ovariatomy.... 16
Lithotomy supra-pubic.... 230
Liver, functions of.... 193
Lizzard's, fossil.... 24
Lobelia.... 146
Local annæsthetic, for gums.... 279
Locusts.... 280
Logwood extract, a disinfectant.... 126
Longevity of man.... 252
Lumbrocoides.... 160
Lying in woman, when to get up.... 305

M.

Macrotys.... 147
Malarial headache.... 34
Malformation, case of.... 2
Malleability of gold.... 134
Malarial fevers, cause and tratment.... 284
Mammary abscess, bandaging for.... 21
Mammites, chronic.... 161
Marine distress signal.... 224
Masturbation.... 303
Matico syrupus, et cort, etc.... 23
Materialistic psychology....163 203
Maternal impressions.... 228
Medical Association of Georgia.... 125
Medical colleges.... 226
Medical colleges, association of.... 137
Medical Congress, international.... 101
Medical Examining Board.... 109
Medical progress and medical journals.... 1

Medical societies........................ 225
Medical schools.......................... 253
Medical swindler......................... 281
Membranous croup, new treatment ....... 265
Menopause................................ 267
Menses, suppression of................... 192
Menstruation in pregnancy............... 313
Menstrual period, epileptic seizures at....... 51
Mercurial vapor bath ..................... 246
Mercury, tonic doses of .................. 222
Meteoric stone........................... 193
Meteoralogical observations............... 52
Microscope............................52 193
Microscopical Society, American........... 83
Mica..................................... 308
Miliaria................................. 115
Milk for babies.......................... 130
Milk beer................................ 162
Milk punch............................... 222
Milk, secretion of, to check.............. 336
Mistura intermitti.......................... 4
Mistura fusca............................... 4
Mistura tuttii.............................. 4
Monobromated camphor, in summer complaints ................................ 226
Monobromated camphor, antagonistic to strychnia.................................. 46
Moody, Dr. J. F., to the country practitioner.................................. 225
Morphia, hypodermic injections of.......... 238
Morphia, subcutaneous, for sciatica, etc..... 202
Morphia, subcutaneous with atropia........ 242
Mouth wash............................... 280
Mucilage of accacia........................ 22
Mutton broth............................. 187
Myrrh, medical uses of..................... 49

N.

Nasal catarrh.........................17 275
Nasal catarrh, new treatment of........... 217
Nasal discharges, vasaline in.............. 137
Naso-phrangeal catarrh ................... 128
Negroes and yellow fever.................. 282
Nephritis, acute.......................... 162
Nerve tonic and alterative.................. 24
Nerve stretching in tetanus................. 11
Nervine.................................... 24
Nervine and anti-spasmodic................. 23
Nervous debility.......................... 190
Nervous insomnia.......................... 153
Nervous diseases, increase of............... 80
Neuralgia................................... 3
Neuralgia, chloral locally for ............ 156
Neuralgia, cerebral......................... 51
Neuralgia, epileptiform.................... 228
Neuralgia, facial......................73 277
Neuralgia, encalyptus in.................... 7
Neuralgia, obstinate........................ 51
Neuralgia of female urethra................ 188
Neuralgia, ovarian.......................... 51
Neuralgia, gelseminum in .................. 228
Neuralgia, supra-orbital (syphilitic)........ 51
Neuralgia, supra-orbital, uncomplicated ..... 61
Neuralgia, wet blanket pack for............ 174
Neuralgia, uterine.......................... 51
Neuralgic pill............................. 133
Newcomer, Dr. D., of Ills., on teratology.... 285
Newton, Dr. O. E. on carbuncle, whitlow and ingrowing of nail........................ 255
Newton, Dr. O. E. on menstruation in pregnancy.................................... 313
Nipples, sore, propylactic for.............. 48
Nitrate of silver, dose and mode of administering.................................. 272
Nitric acid in hoarseness................... 78
Nitric acid in cough....................... 147
Nitric acid, Senna, and Taraxicum......... 335
Nitroglycerine.............................. 82
Nitrous oxide gas.......................... 130
Nutritive enemas.......................... 130
Nux vomica in opium poisoning............. 243

O

Oat meal gruel............................ 187
Obstetric forceps, use of.................. 183
Obstetric memoranda........................ 92
Oesophagus, extracting foreign bodies from.. 19
Oenethria bienis............................ 52
Oil, Harlem................................. 23
Onion, raw, as a diuretic................... 71
Opacities of the eye....................... 159
Opium, pill composita ....................... 4
Opium eating, sudden checking of........... 50
Opium, modified........................... 192
Opium poisoning, nux vomica in............ 252
Opium poisoning, electricity and belladonna in 5
Opium poisoning, vinegar in................ 252
Opium antidotes .......................... 137
Opthalmia strumous......................... 79
Orchitis treaatment of...................... 21
Orchitis, nitrate of silver for.............. 99
Os uteri, ulcers of, cromic acid for........ 131
Osteoclast................................ 137
Ovariotomy, antiseptic method in............ 16
Ovarian nuralgia............................ 51
Ovaries, do they contain male and female ova 157
Oxygen, something new about.............. 220
Oxygen in the sun......................... 252
Oxalate of lime........................... 222

P

Painkiller, examination of.................. 12
Painful micturition......................... 24
Paste, cheap and good..................... 250
Paste, useful recipe for................... 222
Patent humbugs............................ 278
Patent nostrums........................... 305
Perineal abcess and fistula of urethera...... 188
Peritonitis ............................... 163
Pertussis, remedy for...................... 276
Pertussis, salacylic acid in................ 335
Perry Davis, painkiller..................... 12
Petrifaction.............................. 252
Petit, Dr. A., on bleeding at the cord ....... 13
Petroleum in cutaneous affections........... 14
Pharmaceutical and chemical science........ 170
Pharyngitis folicular...................... 134
Pharaoh's serpents, how made.............. 308
Pharyngitis, chronic, tonic for.............. 79
Philadelphia Co. Medical Society, proceedings of..................................... 7
Phlegmasia dolens......................... 205
Phospate of zinc.......................... 277
Phosphates................................ 224
Phosphorus................................ 193
Phosphorated oil, emulsion of................ 48
Phosphorus, solution of in glycerine ....... 22

Phosphorus, emulsion of.................... 79
Phthisis, hypophosphites in................ 249
Phthisis, gallic acid in...... ............... 162
Phthisis, hypophosphites of lime and soda in 162
Piles, cure by puncture.................... 128
Piles, radical cure for.................. 49
Piles, laxative for ... ................. 191
Pilocarpin, effects of hypodermically......... 241
Pilocarpin.......................... 49
Pills, cathartic improved.................. 79
Pill, oxalate of cerium.................. 4
Pill, opii composita.... ........ .......... 4
Pill, podohyllin comp.................... 4
Pityriasis capitis......................... 241
Pityriasis versicolor...................... 95
Placenta previa....... ......... ....... 127
Plant anaesthesia.......... .......... .... 193
Planet, Jupiter.......................... 134
Planet, Mars... ......................... 280
Plaster of Paris bandage..................... 317
Plural births .. ......................... 107
Pneumonia, croupous...................... 296
Pneumonia, to abort.....................191 297
Pocket case of drugs..................... 164
Podophyllin, how to give................... 308
Podophylli, pill comp...................... 4
Podophyllin, in hepatic colic... ............ 276
Polk, Dr Chas., on the sulphate of cinchonidia 257
Polk, Dr. Chas. on the glycerite of kephaline ... ...... .......................... 142
Population of the globe..................... 252
Post partem hemorrhage............ ...... 268
Post partem hemorrhage, new treatment in.. 156
Potentella canadensis..................... 226
Potion for respiratory troubles.............. 77
Post partem hemorrhage.................... 13
Powder, giant.......................... 82
Pregnancy, duration of.................... 223
Pregnancy, sign of........................ 210
Privies, disinfectant for.................... 223
Prolapsus recti............... ............ 239
Propylamine.......... ........ ....... .. 46
Professional brotherhood.................. 108
Protagon ............................. 73
Preserved squash.............. ......... 278
Psychology materialistic................... 163
Psoriassis, india rubber solution............ 17
Puerperal, eclampsia...................... 121
Puerperal fever........................... 335
Puerperal hemorrhage...... .. ........... 132
Pulsatilla............ .................. 148
Pulmonary cavities, treatment of........... 299
Puerperal mania.......................... 205
Purpura hemorrhagica...................... 249
Purgative and diuretic..................... 163
Purgative antibilious powder................ 80
Pupil, indications from... .................
Pulv. columbae com......... ........... .. 4

Q.

Quinia, ecbolic action of... ............... 217
Quinine, bromohydrate of................. 13
Quinine in India ......................... 22
Quinine, in small doses.................. ... 322
Quinine, pills of.... ............... ....100 133
Quinine, physiological effects............. 122
Quinine, solution of, for hypodermic use..... 322
Quinine, sulphate ...................... .. 140

R.

Rabies, woorara for......................... 43
Rabbi, Rosenpits, to a medical class......... 194
Radway's Relief, formula of.................. 12
Rash, produced by bromide of potassium.... 243
Ranula, treatment of...................334 73
Receding year............................ 336
Red lotion of hospitals..................... 335
Respiratory brace......................... 266
Retention of urine........................ 243
Rheumatism, acute, treated by salacine.... 75
Rheumatism, treated with salacylate of soda. 53
Rheumatism, cold water subcutaneously for. 76
Rheumatism, acute articular, cynanide of zinc for.............................. 77
Rheumatism, chronic, . .... ............ 106
Rheumatism, hot packing in............. . 78
Rheumatism, cynanide of zinc in........... 305
Rheumatism, muscular..................... 107
Rheumatism, swollen joints from........... 106
Rheumatism, ammonia treatment of........ 258
Rheumatism, chronic....................... 336
Rheumatism, lemon juice in................ 227
Rheumatism, prescriptions for..... .....190 250
Rheumatism, salacylate of soda in........... 78
Rheumatism, veratrum in . ................. 92
Rheumatic arthritis... ..................... 279
Rheumatic arthritis, treatment of.... ...... 124
Rheumatic headache.......... ........... 34
Rhus poisoning.......................... 132
Ricords' cough pills....................... 334
Ringworm ............................... 223
Rum stomach ............... .......... 334

S

Saccharated calomel................ ...... 80
Safrol ......... ....................... 78
Sage's catarrh remedy. .................... 126
Salacine for chills........................ 189
Salacine in rheumatism.................... 15
Salacylic acid, soluble in spirits nitre........ 156
Salacylic acid, caution in use of............. 160
Salacylic acid, concentrated solution of..... 20
Salacylic acid, poisoning by................. 276
Salacylate of soda....... .............. 14
Salacylate of soda as an antipyretic......... 15
Salacylate of soda in rheumatism.........78 53
Salt, death for want of.................... 279
Salt water made fresh...................... 82
Salivation, remedy for...................224 331
Sanitary commission of Atlanta............. 196
Sanguinaria ............................. 146
Santonin................................ 214
Sawdust pads............................ 295
Sawyer's forceps... ...................... 195
Sclerotic acid........................... 332
Scarlatinal albuminuria............. ..... 14
Scalp, eruptive disease of......... ....... 80
Sciatica........................... ... 3
Sciatica, deep injection of chloroform in... 127
Sciatic neuralgia......................... 94
Scraps of practice......................... 307
Scrofula, formula for....................... 106
Scrofula, ulcers.......................... 218
Sebaceous glands, to destroy............... 223
Sedatives, for the young................... 221
Sedative antiseptic........................ 222

Seed, 1,600 years old.................... 82
Sexual hygiene.......................... 274
Seven Springs mass...................... 170
Shaving soap............................ 334
Shoulder presentation, version by the vertex 118
Sick headache........................... 33
Sick stomach............................ 155
Sick stomach, hot water for............. 129
Sideraphite............................. 24
Sign of early pregnancy................. 210
Skin, eruptive diseases of.............. 115
Skin, cracked........................... 107
Skin affections, remedy for............. 80
Skin, brown discoloration of............ 223
Slippery elm jelly and tea.............. 223
Smith, Dr. A. A., on therapeutics of the head 32
Small doses of medicine, value of....... 270
Smallpox, important if true............. 53
Snails, vitality of..................... 193
Snake bite, carbonate of amonia in...... 223
Snake bite, to cure hydrophobia......... 244
Sneezing to reduce hernia............... 95
Soda for burns.......................... 274
Soda, hypophosphate of.................. 14
Sore throat, chronic.................... 134
Sore nipples.......................48 224
Soothing syrup, Winslow's............... 305
Sozodont................................ 305
Spectacles, colored..................... 77
Spleen.................................. 303
Spermatorrhea........................... 133
Spray carbolic, in bronchial catarrh.... 76
Steel, to take rust from................ 249
Sticta pulmonaria....................... 147
Stillingia.............................. 147
Stimulants in typhoid fever............. 69
Still burning—a coal mine............... 193
Stove blacking.......................... 106
Strumous ophthalmia..................... 79
Strumous cases, remedy for.............. 24
Strychnia.........................46, 250
Strictures.............................. 217
Subcutaneous injection of morphia....... 202
Sugar-coated pills...................... 198
Sugar-coated pills, originator of....... 310
Sulphate of cinchonidia, &c.........257, 338 139
Sulphate of zinc in chorea.............. 160
Sulphocyanide of potassium.............. 103
Sulphuric acid in chronic urticaria..... 156
Sulpho-phenate of soda.................. 276
Summer complaint, baths in.........41 273
Sun..................................... 52
Sunlight................................ 193
Suprapubic lithotomy.................... 230
Supra orbital neuralgia, syphilitic..... 51
Supra orbital neuralgia, uncomplicated.. 51
Sweating, coliquitive................... 71
Sweating, profuse....................... 216
Sweet spirits of nitre dissolves salacylic acid 156
Swift, Dr. T. B., letter from........... 226
Sylicate of soda in sciatica............ 224
Syphilis, curability of................. 132
Syphilis, treatment of.................. 42
Syphilis, fumigation in................. 314
Syphilis, doubtful points in history of. 60
Syphilis and scrofula................... 244
Syphilitic fathers and healthy children. 329
Syphilitic headache..................... 34
Syrupus assafœtida...................... 4
Syrupus Pectoralis...................... 4
Syrupus asthmaticus..................... 4
Syrupus matico et cort granata.......... 23

T.

Tæniafuges, combination of.............. 75
Tanniun gargle.......................... 336
Tape worm.........................278 336
Tape worm, electuary for................ 132
Tartar emetic........................... 146
Tar water in typhoid fever.............. 241
Tax on physicians....................... 195
Taxis, Dugas' method of................. 123
Taynya, new remedy for syphilis and scrofula 244
Teeth, sympathetic lesion, etc.......... 203
Telephone......................107 280 363
Temperature of the earth................ 234
Testicles and the procreation power..... 280
Tetanus, nerve stretching in............ 11
Tetanus, traumatic case of.............. 262
Teratology.............................. 285
Texas, medical convention............... 138
Thermometry in diseases................. 104
Thigh, antiseptic trsatment of.......... 10
Thompson's eye water.................... 221
Throat, chronic sore.................... 134
Tic doloreux, bromide of potassium in... 73
Tinia, tonsurans........................ 81
Tinnitus aurium, hydrobromic acid in.... 248
Toast water............................. 187
Tobacco................................. 282
Toe itch................................ 162
Tooth powder............................ 160
Tomato leaf, singular property of....... 185
Tonic, a fine........................... 250
Tonic doses of mercury.................. 221
Tonic, laxative and anodyne............. 335
Tong pang chong......................... 240
Toothache............................... 107
Toothache drops....................224 221
Tonsils, enlarged....................... 335
Tonsils, pultaceous concretion from..... 289
Tortion in hemorrhage................... 15
Tracheotimy to remove foreign body...... 58
Tracheotimy with the thermo cantery..... 131
Trepanning for fracture................. 215
Transactions, society of California..... 196
Transactions medical society of Virginia 83
Transactions Miss. Med. Ass............. 84
Transactions medical society of Alabama. 181
Transfusion in anæmia................... 254
Transfusion and auto-transfusion........ 37
Trismus nascentium...................... 257
True physician, marks of................ 282
Tuberculosis, transmissible............. 238
Turkish bath described.................. 85
Turpentine inhalations.................. 336
Typhoid fever, ergot in............76 44
Typhoid fever, how produced............. 125
Typhoid fever, iodoform in.............. 332
Typhoid fever, reduction of heat in..... 274
Typhoid fever, stimulants in............ 69

U

Ulcer gastric, milk treatment........... 47
Ulcers, chloral in...................... 50
Ulcers corneal.......................... 159
Ulcers, scrofulous, plaster for......... 218
Ulcers of leg, bandaging for............ 151

Umbilical hernia in infants................ 49
Union by first intention.................... 148
United States Pharmacopea................ 83
Upper Georgia, climate of.................. 55
Uræmic headache.......................... 34
Uræmia convulsions, &c................... 245
Urates........................................ 222
Urate of lime............................... 222
Urate of ammonia........................... 224
Urethra, female, neuralgia of............. 188
Urine, retention of......................... 243
Urine, incontinence of in children........ 251
Urinary deposits.........................161 251
Urticaria, sulphuric acid in............... 156
Uterus dilatation of........................ 70
Uterus, irreducible retroflexion of........ 57
Uterine disorders........................24 23
Uterine neuralgia........................... 51
Uterine injections, unsafe.................. 125
Uterine dilator.............................. 195

V

Vanderbeck, Dr. C. C., current neurological matters........................................ 201
Vanderbeck, Dr. C. C., treatment of nervous diseases, &c.................................3 228
Valuable prescriptions...................... 51
Vaccine and erysipelas...................... 332
Varices of the leg........................... 186
Varieties in animals......................... 107
Vasaline..................................... 277
Vasaline in nasal discharges............... 137
Varicocile, six cases of.................... 45
Vegetable diet for epileptics............... 50
Velpeau's diarrhoea mixture................ 23
Veratrum, gelsominum as an antidote for... 40
Veratrum poisoning......................... 301
Veratrum viride............................. 96
Veratrum viride, in puerperal convulsions... 99
Veratrum viride, in rheumatism............ 92
Venous pulse from chloroform.............. 52
Vermin...................................... 335
Vertebrated animals in the human stomach.. 54
Version by the vertex in shoulder presentation......................................... 118
Vertigo, hydrobromic acid in............... 306
Vesical catarrh............................. 191
Vesical disturbances from deptheria........ 14
Vesical hemorrhage......................... 131
Vinegar in opium poisoning................ 252
Vomiting after debauch..................... 270
Vomiting in cholera infantum.............. 180
Vomiting in phthisis........................ 270
Vomiting in pregnancy..................... 105
Vomiting, obstinate......................... 224
Vomiting, with children.................... 270

W

Warts, cromic acid for..................... 77
Water....................................... 225
Water, influence on biliary secretion...... 327
Wet blanket in fever, dysunnorrhea, &c..... 174
White-head torpedo......................... 252
Whitlow..................................... 256
Whitlow of the thumb...................... 329
Wild yam, or deascoria..................... 152
Wild cherry, comp. syrup of................ 333
Wild cherry bark elixir of.................. 334
Wine bitters................................ 133
Wine whey.................................. 223
Wilson, Dr. J. S., on Turkish bath......... 85
Wizzard oil, formula of..................... 12
Wonders of a hen's egg.................... 82
Woodruff scientific expedition............. 196
Woorara in rabies.......................... 43
Woodward, Dr. on eucalyptus.............. 7
Word, Dr. R. C., medical progress and med. journals..................................... 1
World's hair restorer....................... 305
Worm lozenges.............................. 305
Worms...................................... 188
Wounds, treatment of....................... 259

X

Xanthium strumarium, or cocklebur........ 124

Y

Yellow fever................................ 278
Yellow fever and negroes.................. 282
Yerba santa................................ 176

